# Perception of quality health care delivery under capitation payment: a cross-sectional survey of health insurance subscribers and providers in Ghana

**DOI:** 10.1186/s12875-018-0727-4

**Published:** 2018-03-07

**Authors:** Francis-Xavier Andoh-Adjei, Eric Nsiah-Boateng, Felix Ankomah Asante, Ernst Spaan, Koos van der Velden

**Affiliations:** 1 NHIA 36-6th Avenue, Ridge. PMB Ministries Post Office, Accra, Ghana; 2 Institute of Statistical, Social and Economic Research (ISSER) University of Ghana, Legon-Accra, Ghana; 3 Radboud Institute for Health Science, Department for Health Evidence, Radboud University Medical Center, Nijmegen, Netherlands; 4 Radboud Institute for Health Science, Department for Primary and Community Health, Radboud University Medical Centre, Nijmegen, Netherlands

**Keywords:** Capitation payment, Perceived quality of care, Health care providers, Health insurance subscribers, Ghana

## Abstract

**Background:**

Ghana introduced capitation payment method in 2012 but was faced with resistance for its perceived poor quality of care. This paper assesses National Health Insurance Scheme subscribers and care providers’ perception of quality of care under the capitation payment method.

**Methods:**

This is a cross-sectional survey of subscribers and care providers perception of quality of care in three administrative regions of Ghana using a 5-point Likert scale for the assessment based on a set of quality of care measures. We performed descriptive analysis to determine average perception of quality of care scores for each of the measures used. Bivariate and multivariate analyses were also performed to examine relationships between respondent’s characteristics and their perception of quality of care.

**Results:**

In general, subscribers expressed positive perception about the quality of care though subscribers in Ashanti were less positive compared to those in the Central region. A chi-square analysis, however, showed significant differences in subscribers’ perception of quality of care by occupation (*p* = 0.002), region (*p* = 0.007) length of NHIS membership (*p* = 0.006), and age (*p* = 0.014). Multivariate logistic regression analysis also showed that different factors, other than region of residence, were significantly associated with perceived good quality of care. Analysis of health care providers’ responses also showed significant differences in their perception of quality of care by region (*p* = 0.001). Multivariate logistic model showed that health care providers in the Volta region (OR = 0.14, 95% CI: 0.03–0.58) were significantly less likely to perceive quality of care as good compared to those in the Ashanti region.

**Conclusion:**

Subscribers and care providers across the three regions have relatively good perception of the quality of health care in general though subscribers in Ashanti were less positive than those in the Central region. It is, therefore, plausible that capitation payment may have influenced the relatively low perception of quality of care in the Ashanti region.

**Electronic supplementary material:**

The online version of this article (10.1186/s12875-018-0727-4) contains supplementary material, which is available to authorized users.

## Background

Ghana introduced a National Health Insurance Scheme (NHIS) in 2003 to provide financial health protection against the cost of health care services for the population. Initially, the National Health Insurance Authority (NHIA) applied fee-for-service (FFS) method for the payment of its credentialed providers but had to introduce diagnosis-related-grouping (DRG) payment with the view to addressing observed increases in utilization and claims expenditures [1]. Years into the implementation, the DRG was found to have further contributed to cost escalation, almost tripling the claims expenditure made under the fee-for-service dispensation. After careful consideration of the issue, the NHIA decided to introduce capitation payment for primary out-patients’ services beginning with a pilot in the Ashanti region of Ghana. The decision to pilot capitation payment in the Ashanti region of Ghana, however, engendered various reactions from providers as well as politicians and civil society groups. In a press release carried by the Ghanaian Times newspaper of 02/01/2012, the Chairman of the Association of Private Medical and Dental Practitioners was quoted to have stated that *“the system (capitation payment) is detrimental to quality health care provision and a major threat to the survival of private health facilities”*. This was followed by a publication in the Daily Guide newspaper of 25/01/2012 which quoted the President of the Ashanti Development Union (ADU), a civil society group, as saying *“information reaching my office indicates that since the implementation of the (capitation) policy in the region three weeks ago hospital attendance has gone down considerably”* a situation that suggests perceived poor quality of care on the part of health insurance subscribers in the region following the introduction of the capitation payment policy [2]. With the on-going debate on capitation payment and it’s perceived negative effects on care provision, it is instructive to assess the perception of both users and providers of health care services about the quality of care provision. Haddad et al. note that assessment of quality of primary care could be enhanced by including patients’ perceptions as well as professional judgment of quality [3]**.** Patients’ perception of service quality, in particular, has become a vital aspect of quality assessment of health care because of the relationship between health care utilization and user perception of quality [4, 5]. Mack et al. are of the view that “without a direct assessment of patients’ values”, policy makers “may create standards of care that meet physicians’ values better than those of patients” [6]; and even though patients’ perception of quality may not necessarily be in conformity with professional standards of quality an exploration of patients’ perception could yield useful information for improving quality care delivery [3]. Studies on Ghana’s capitation payment sighted in literature focused on knowledge, perception and expectations of insured clients [7], subscriber-provider trust relationship [8], the challenges associated with the implementation of the capitation payment in the Ashanti region [9] and capitation payment and membership retention [10] . We are yet to find any specific study on NHIS’ provider payment method and how that influence subscribers and providers’ perception of quality of care. The objective of this study was, therefore, to assess NHIS subscribers and credentialed providers’ perception of quality care under the capitation payment regime in the Ashanti region alongside those of two other regions that are yet to implement capitation payment in order to establish whether capitation payment influences subscribers and health care providers’ perception of quality care in the Ashanti region. A better understanding of both subscribers and providers perspectives having real-world experience of capitation payment in primary care delivery, will contribute to the on-going debate and provide guidance for the NHIA as they plan to scale up capitation payment countrywide. It will equally provide guidance to other low/middle-income countries considering capitation payment as a provider payment method in their health insurance scheme and also add to existing literature on factors that influence quality of care perception of both healthcare providers and users of healthcare services.

## Methods

### Study design

We adopted a survey design for the study. Since no baseline study was done prior to the implementation of the policy in the Ashanti region to enable comparison in order to determine the effect of the capitation payment on subscribers and providers’ perception of quality care in the Ashanti region, we adopted the post-test-only with non-equivalent group design [11] and purposively selected Volta and Central regions that were being prepared for the next implementation phase as “control” regions to enable the establishment of any relationship between capitation payment and clients/providers’ perceived quality of care in the Ashanti region. We adopted perceived quality of care frameworks, several of which have been developed to help assess users and providers of health care services’ perception of quality care delivery [5]; [12]; [13]; [14, 15], to guide our study. In their description and development of a 16-item scale to measure perceived quality in India, Rao et al. [4] identified five distinct dimensions of perceived quality, namely (i) medicine availability, (ii) medical information given to patients by their physician (iii) staff behavior towards patients, (iv) doctors’ interpersonal behavior such as their responsiveness to the patients’ concerns, and (v) the hospitals’ infrastructure such as cleanliness and availability of other amenities). Amala de Silva [16] also identified dimensions of quality as dignity, autonomy, confidentiality, prompt attention, quality of basic amenities, access to social support networks during care and choice of care provider. Drawing on these and other existing frameworks we developed an 18-item scale and 12-item scale to assess the perception of subscribers and providers, respectively, on quality of care under capitation payment regime. The item scales were clustered around six key dimensions: (i) staff availability and prompt attention; (ii) dignity and respect; (iii) confidentiality; (iv) service quality; (v) communication; and (vi) accommodation/cleanliness.

### Research setting

The study took place in three regions of Ghana: Ashanti, Volta and Central regions in 2014. The Ashanti region is the first among the 10 regions in Ghana to begin the implementation of the capitation payment policy in 2012, and was therefore selected as the “intervention” region for the study. The policy will be implemented in the Volta region from 2016 and in the Central region in 2017 in line with NHIA’s implementation plan. The two control regions have about the same socio-demographic characteristics, about the same level of NHIS active card-bearing members and about the same number of credentialed health care providers. Table 1 below presents detailed information on the basic socio-demographic characteristics of the three regions.

**Table 1:** Basic socio-demographic and health service/NHIS indicators

Indicator	Ashanti	Volta	Central	National
a. Socio-demographics (GSS 2013)				
Percentage polpulation (2010 PHC)	19.4	8.6	8.9	24,658,823
Economically active population (2010)	19.1	8.4	8.6	43.9
Percentage population employed (2010)	18.6	8.6	8.5	
Percentage self-employed of the employed	65.5	75.3	69.2	64.9
Population density (km^2^) (2010)	196	103	224.1	103
Percentage urban population (2010)	60.6	33.7	47.1	50.9
Sex ratio (males/100 females) (2010)	94	92.8	91	95.2
Households (2010)	1,126,216	495,603	526,764	5,467,136
Average household size (2010)	4.1	4.2	4.0	4.4
Percentage population literate (2010)	82.6	73.5	78.2	74.1
b. NHIS service availability (NHIA, 2013)				
NHIA District Offices	25	15	13	166
Active NHIS card-bearing members	1,715,174	910,559	866,831	10,145,196
Percentage active to regional population	34	28	23	
NHIS-credentialed service providers (2013)	619	321	334	3832
c. Health personnel availability (GHS 2013)				
Percentage share of health professionals	18.2	8.5	8.6	
Percentage share of nurses (Professional)	45.5	53.4	39.3	
Percentage share of nurses (Enlrolled)	54.5	46.6	60.7	
Number of Doctors	96	36	26	n/a
Number of Community Health Nurses	157	264	130	n/a

Sources: GSS 2010 Population and Housing Census, 2013. Available at www.statsghana.gov.gh/.../2010_POPULATION_AND_HOUSING_CENSUS_FINALNHIA Annual Report, 2013. Available at www.nhia.gov.gh/nhia.aspx; GHS Annual Report 2013. Available at www.ghanahealthservice.org/.../Ghana_Health_Service_2014_Annual_Report

### Population and sampling

#### Subscriber sampling

Multi-stage sampling was applied to recruit subjects for the survey. The list of Enumeration Areas (EAs) used for the 6th round of the Ghana Living Standard Survey (GLSS6) was used as the sample frame. We adopted the sample design used by the Ghana Statistical Service (GSS) for post enumeration survey (PES) to determine the number of EAs (clusters) in the region. In the said design, each of the ten regions in Ghana was treated as domain for selection and analysis, and was based on probability sample of 250 EAs of which 45 were in Ashanti, 23 in Volta and 19 in Central regions. Thus, a total of 87 clusters in the three regions were randomly selected at the first stage of the sampling process. We then used the WHO revised Expanded Programme of Immunization (EPI) survey reference manual [17] as guide for determining the sample size per EA. We opted for the desired precision level of ±3% and expected coverage of 95% to determine the number of subjects for the survey. On the basis of the WHO immunization coverage cluster survey reference manual, we arrived at 10 subjects per cluster (EA) for Ashanti giving a representative sample of 450 households (subjects), 18 subjects for Volta giving a sample of 414 households and 21 clusters for the central region giving a sample of 399 household (Additional file 1). We therefore selected a total of 1, 263 samples for the study. We then sought the assistance of personnel of the Ghana Statistical Service to draw a list of the households and their replacements which was given to the interviewers for each of the 3 regions for the field interviews. In each household an adult card-bearing member aged 18 years and above was randomly selected and interviewed.

#### Provider sampling

We used the G-power analysis programme (G* Power 3.1) [18, 19] to determine the appropriate sample size of credentialed providers. We assumed an effect size of 0.4, an alpha (α) of 0.05 and beta (1-β) of 0.80; and allocation ratio (N2/N1) of 1.1. The outputs were 200 samples: 95 for group one (intervention region) and 105 for group two (the control regions). Based on the total number of NHIS-credentialed providers in the two control regions as at the end of year 2013, we proportionally allocated 48% of the samples for the control regions to Volta and 52% to Central, respectively.

#### Data collection method

We administered closed-ended questionnaires on 200 credentialed providers and 1263 subscribers in the three regions using face-to-face interview. The questionnaire focused on socio-demographic characteristics of respondents and their perception of quality of care under capitation payment regime. We asked both subscribers and healthcare providers to score the quality of care statements on a 5-point Likert scale of don’t know (0); strongly disagree (1); disagree (2); agree (3); and strongly agree (4) Additional file 2.

#### Data analysis

First, we performed a principal component analysis (PCA) to transform the set of statements for assessing quality of care into five uncorrelated components of dignity and respect, confidentiality, service quality, communication, and accommodation/cleanliness [4]; [5]; [12]; [13]; [14]; [15]; [16]. The factor scores obtained from this analysis were used to estimate mean perception score for each respondent. Respondents who obtained positive mean perception score were assigned to “perceived good quality of care” group and those who obtained negative mean score were assigned to a “perceived poor quality of care” group. Subsequently, we performed a Chi-square test to determine relationship between respondents’ socio-demographic characteristics and the binary outcome variable “perception of quality of care” (perceived good quality of care = 1; perceived poor quality of care = 0). The bivariate analysis was conducted to test independent associations between each independent variable and the outcome variable, and to only advance those that show statistical significance at *p* < 0.10 to a multivariate logistic regression model for further analysis (http://www.populationsurvey.com/). In the first multivariate logistic regression model to determine associations between the independent variables and subscribers “perceived good quality of care”, *gender, education level, and card used in past 2 years* were included in the model despite the set statistical significant threshold for inclusion. In the second model to examine association between the independent variables and healthcare providers “perceived good quality of care”, *age, gender, primary status at health facility, years in practice, health facility ownership and health facility* were included in model. We decided to include all the eight independent variables in the healthcare providers’ model, having noted in literature [20, 21] that all the variables listed had some relationship with quality of care.

We also examined the total variance in respondents’ perception of quality of care delivery explained by the components extracted. As a criterion, only components with eigenvalue of one or more were retained. That is, we dropped any component that accounted for less variance than did a single question or variable. Varimax rotation with Kaiser Normalization was then employed to minimize the complexity of the components by making the large loadings larger and small loadings smaller within each component. The PCA produced a Kaiser-Meyer-Olkin (KMO) measure of sampling adequacy of 0.813 and Bartlett’s test of sphericity of *p* < 0.001 for the 18 statements for assessing subscribers perception of care and 0.770 and *p* < 0.001 for the 12 statements for healthcare providers’ perception of quality of care delivery. The proportion of variance explained by each component for subscribers’ and healthcare providers’ perception of quality of care are attached as Additional files 3 and 4, respectively. The analyses were performed using Stata version 13 and SPSS version 20 softwares and the results summarized in tables.

## Results

### Relationship between subscribers’ characteristics and perceived good quality of care

One thousand and ninety-nine (1099) subscriber respondents participated in the survey, representing 87% response rate. About 56% of respondents were above the average age of 44.31 years (SD = 17.68), 78% were females, and 35% were in the Ashanti region. Fifty-five per cent of the respondents resided in the rural areas, 63% were married, 40% had middle or junior high school education, and 62% were self-employed. About 38% of the respondents had been members of the NHIS for less than one year while 23% had been with the scheme for four years or more; 90% had used the card for the past two years prior to the survey, and 43% had attended health care facility between one and three months before. Table 2 summarized relationship between socio-demographic characteristics of subscribers and perceived good quality of care. In general, 55.5% (610) of subscribers expressed positive perception about the quality of care. In terms of individual regions, 53% of subscribers in Ashanti region perceived quality of care as good compared to 51% and 62% in the Volta and Central regions, respectively. There were significant differences in subscribers’ perception of quality of care by occupation (*p* = 0.002), length of NHIS membership (*p* = 0.006), region (*p* = 0.007) and age (*p* = 0.014). Results of the multivariate regression analysis showed that subscribers aged 44 years and above were significantly more likely to rate their perception of quality of care as good compared to those below 44 years (OR = 1.45, 95% CI: 1.06–1.99) (Table 3). However, elementary workers (OR = 0.27, 95% CI: 0.04–0.98) and Technicians (OR = 0.20, 95% CI: 0.04–0.98) were significantly less likely to rate their perception of quality of care as good relative to a senior officer/manager. Similarly, subscribers with four or more years of enrolment in the scheme were less likely to rate their perception of quality of care as good compared to those with less than a year of enrolment (OR = 0.68, 95% CI: 0.46–0.99).

**Table 2:** Association between subscribers’ characteristics and perceived good quality of care

Characteristic	N	%	X^2^	*p*-value
Age			5.9785	0.014
< 44	332	52.3		
44+	278	59.8		
Gender			1.5658	0.211
Male	144	59.0		
Female	466	54.5		
Region			9.8067	0.007
Ashanti	201	52.5		
Volta	181	51.2		
Central	228	62.1		
Setting			3.7906	0.052
Urban	261	52.3		
Rural	349	58.2		
Marital status			4.6840	0.456
Married	380	55.3		
Separated	21	56.8		
Divorced	20	42.6		
Widowed	91	58.0		
Cohabitation	14	66.7		
Never married	84	56.0		
Education level			2.8357	0.586
Primary	114	59.1		
Middle/JHS	240	54.7		
Secondary/SSS	78	57.0		
Higher/Tertiary	31	47.8		
Never attended school	147	55.5		
Occupation			22.7185	0.002
Senior officer/manager	11	73.3		
Professional	19	42.2		
Technician	6	40.0		
Services/sales worker	92	51.1		
Agriculture	141	60.5		
Plant/machine	1	25.0		
Elementary worker	91	46.4		
Other	249	60.6		
Length of NHIS			14.3779	0.006
membership (yrs)				
< 1	259	62.1		
1	45	54.9		
2	79	54.1		
3	103	53.1		
> =4	124	47.7		
Card used in past			2.1702	0.141
2 years	66	62.3		
No	544	54.5		
Yes				
Total	610	55.5

**Table 3:** Multivariate logistic regression model for subscribers perceived good quality of care

Characteristic	OR	Std. Err.	z	P > z	[95% C.I]
Age (yrs)					
< 44	1.00				
44_+_	1.45	0.23	2.33	0.020	1.06–1.99
Gender					
Male	1.00				
Female	0.85	0.14	−0.89	0.371	0.60–1.20
Region					
Ashanti	1.00				
Volta	0.82	0.13	−1.17	0.243	0.59–1.14
Central	1.36	0.22	1.84	0.065	0.98–1.89
Setting					
Urban	1.00				
Rural	1.19	0.15	1.36	0.175	0.92–1.55
Marital status					
Married	1.00				
Separated	0.96	0.33	−0.10	0.917	0.48–1.92
Divorced	0.53	0.17	−1.94	0.052	0.28–1.00
Widowed	0.85	0.18	−0.70	0.484	0.56–1.31
Cohabitation	1.29	0.62	0.53	0.599	0.49–3.34
Never married	1.03	0.20	0.17	0.868	0.69–1.52
Highest level of education					
Primary	1.00				
Middle/JHS	0.80	0.14	−1.18	0.237	0.56–1.15
Secondary/SHS	0.85	0.21	−0.62	0.534	0.52–1.38
Higher/Tertiary	0.55	0.18	−1.78	0.074	0.28–1.06
Never attended school	0.73	0.15	−1.51	0.132	0.49–1.09
Occupation					
Snr officer/manager	1.00				
Professional	0.27	0.18	−1.92	0.055	0.07–1.03
Technician	0.20	0.16	−1.98	0.048	0.04–0.98
Services/Sales	0.33	0.20	−1.76	0.078	0.09–1.13
Agriculture	0.40	0.25	−1.45	0.148	0.12–1.37
Plant/Machine	0.12	0.16	−1.59	0.111	0.01–1.62
Elementary worker	0.27	0.17	−2.08	0.037	0.08–0.92
Other	0.40	0.24	−1.47	0.143	0.12–1.35
Length of NHIS membership (yrs)					
< 1	1.00				
1	0.72	0.19	−1.20	0.230	0.43–1.22
2	0.79	0.17	−1.07	0.283	0.52–121
3	0.78	0.15	−1.23	0.218	0.53–1.15
> =4	0.68	0.13	−2.00	0.046	0.46–0.99
Card used in past two years					
No	1.00				
Yes	0.80	0.17	−0.97	0.331	0.52–1.24
No. of obs	1099				
LR chi2 (15)	55.17				
Prob > chi2	0.0007				
Log likelihood	− 727.51048				
Pseudo R2	0.03665				

### Relationship between healthcare providers’ characteristics and perceived good quality of care

One hundred and seventy-three (173) health care providers participated in the survey, and a response rate of 86% was achieved. Half of the respondents were females; average age was 44.30 years (SD = 11.33); 42% were in Ashanti region; and 70% were in the urban setting. Respondents’ average years in practice was 11.18 years (SD = 9.62). Forty per cent (40%) occupied positions other than medical officer, medical assistant, and nurse-in-charge; 65% worked in quasi-government health care facilities; and 78% worked at the hospital level. Results of the Chi-square analysis showed that 80% (139) of healthcare providers perceived quality of care delivery as good (Table 4). There was significant difference in their perception of quality of care by region (*p* = 0.001). Eighty-six percent of healthcare providers in Ashanti region perceived quality of care as good compared to 63% and 89% in Volta and Central regions, respectively. The multivariate logistic regression revealed that being a health care provider in the Volta region OR = 0.14, 95% CI: 0.03–0.58) significantly decreases the likelihood of rating perception of quality of care as good compared to being in the Ashanti in region (Table 5). Likewise, healthcare providers at the hospital level (OR = 8.11, 95% CI: 1.05–62.07) were significantly more likely to perceive quality of care as good compared to those at the health centre level.

**Table 4:** Association between care providers’ characteristics and perceived good quality of care

Characteristic	N	%	X^2^	p-value
Age			0.0143	0.905
< 44	67	8072		
44+	72	80.0		
Gender			2.2292	0.135
Male	73	84.9		
Female	66	75.9		
Region			13.5273	0.001
Ashanti	62	86.1		
Volta	30	62.5		
Central	47	88.7		
Setting			0.8579	0.354
Urban	95	78.5		
Rural	44	84.6		
Primary status at facility			3.7071	0.447
Medical officer	26	78.8		
Medical assistant	14	66.7		
Nurse-in-charge	42	85.7		
Other	57	81.2		
Years in practice			1.2596	0.262
< 11	88	77.9		
11+	51	85.0		
Facility ownership			1.8567	0.395
Quasi-government	50	76.9		
Mission	44	78.6		
Private	45	86.5		
Facility type			4.9394	0.294
Health centre	4	66.7		
Clinic	17	80.9		
Maternity home	3	60.0		
Hospital	111	82.8		
CHPS	4	57.1		
Total	139	80.4

**Table 5:** Multivariate logistic regression model for care providers perceived good quality of care

Characteristic	OR	Std. Err.	z	P > z	[95% C.I
Age (yrs)					
< 44	1.00				
44+	0.90	0.48	−0.19	0.846	0.31–2.56
Gender					
Male	1.00				
Female	0.46	0.24	−1.48	0.138	0.16–1027
Region					
Ashanti	1.00				
Volta	0.14	0.10	−2.70	0.007	0.03–0.58
Central	1.27	0.82	0.37	0.709	0.35–4.55
Setting					
Urban	1.00				
Rural	2.90	1.61	1.91	0.057	0.97–8.66
Primary Status at facility					
Medical Officer	1.00				
Medical Assistant	1.01	0.81	0.02	0.987	0.20–4.92
Nurse in-charge	2.53	1.95	1.21	0.228	0.55–11.4
Other	0.65	0.49	−0.55	0.579	0.15–2.88
Years in practice					
< 11	1.00				
11+	1.40	0.75	0.62	0.534	0.48–4.04
Facility ownership					
Quasi-government	1.00				
Mission	0.61	0.33	−0.88	0.378	0.21–1.80
Private	1.90	1.17	1.05	0.294	0.57–6.36
Facility type					
Health centre	1.00				
Clinic	2.69	3.13	0.85	0.394	0.27–26.33
Maternity home	0.82	1.27	−0.13	0.899	0.03–17.13
Hospital	8.11	8.42	2.02	0.044	1.05–62.07
CHPS	.59	.87	−0.35	0.725	0.03–10.48
No. of obs	172				
LR chi2 (15)	32.71				
Prob > chi2	0.0052				
Log likelihood	−69.158398				
Pseudo R2	0.1912				

### Perception of quality of care components and factor loadings

Findings of the PCA showed that five components namely; communication, dignity and respect, service quality, accommodation and cleanliness, and confidentiality were extracted from the 18 statements for assessing subscribers’ perception of quality of care delivery (Table 6) whilst three components (communication, availability and promptness, accommodation and cleanliness) were extracted from the 12 statements for assessing healthcare providers’ perception of quality of care (Table 7). Together, the five out of the 18 components accounted for about 65% of the total variance in subscribers’ perception of quality of care delivery (Additional file 2). Similarly, the three out of the 12 components extracted from the healthcare providers statements accounted for 65% of the total variance in their perception of quality of care delivery (Additional file 3). In both subscriber and provider perception of quality components, communication accounted for much of the perception of quality, 26% and 44.2% respectively. Thus, communication had heavier factor loadings compared to other components. Whilst “dignity and respect” was the second highest component that accounted for 16% variance in the subscribers’ perception of quality, “availability and promptness” was the second highest component, which accounted for 11.6% of variance in healthcare providers’ perception of quality of care delivery.

**Table 6:** Subscribers perception component matrix and factor loadings

Item no.	Statement	Component				
		Communication	Dignity and respect	Service quality	Accommodation and cleanliness	Confidentiality
1	He/she spent time to advise me on preventive care	.870				
2	Prescriber made time to discuss my health condition and treatment	.853				
3	He/she explained everything about treatment	.797				
4	Medicines prescribed were very good	.776				
5	He/she opened up to me for statements about treatment	.757				
6	Nurses treated me with dignity		.943			
7	Prescriber gave me option to accept or refuse treatment		.933			
8	Nurses were cautious towards me		.907			
9	Prescriber made a good diagnosis			.812		
10	Treatment was effective for recovery and cure			.777		
11	I was able to see prescriber within 30 min			.586		
12	There was a prescriber available to attend to me			.551		
13	No congestion at facility during last visit				.738	
14	Seats were enough at facility during last visit				.606	
15	During last visit at facility, environment was clean				.594	
16	During last visit, waiting area was well ventilated				.559	
17	Confidentiality in consulting room					.848
18	Health information is kept secret and confidential					.729

**Table 7:** Providers perception component matrix and factor loadings

Item no	Statement	Component		
		Communication	Availability and promptness	Accommodation and cleanliness
1	We explain to clients how to take their medication	.855		
2	We do proper diagnosis	.812	.345	
3	We make time to advise clients on disease prevention	.805		
4	Clients can ask statements about treatment	.726		
5	Consulting rooms good enough to give privacy	.677		
6	Environment at facility is neat	.675	.350	.396
7	Nurses show interest in clients and makes them feel comfortable	.549		
8	Enough prescribers to look at clients		.877	
9	There is always a prescriber at post		.845	
10	Doctors show interest in clients and makes them feel comfortable	.342	.353	
11	Waiting area is well ventilated			.912
12	Facility has enough seats in waiting area	.454		.632

## Discussion

### Perception of quality of care by subscribers and care providers

In general, our study findings show that both users of health care services and their providers have relatively good perception of the quality of health care delivered given that slightly over 50% of subscribers expressed positive perception of quality; plausible reasons being courtesy bias on the part of those respondents towards their care providers [8]. It is also plausible to attribute the positive ratings by users of the health care services to their limited knowledge of what constitutes quality healthcare [22]. Notwithstanding the relatively positive perception of quality among our study subjects across all three regions, we found that subscribers in the Central region were more positive about the quality of care than those in the Ashanti region whilst those in the Volta region were slightly less positive than those in the Ashanti region, suggesting that region of residence is a factor that influences peoples’ perception of quality care. The higher quality perception rate by respondents in Central region over that of Ashanti region supports findings of a study by Amo-Adjei et al. [2] in which subscribers in the Western region (that shares border with Central region) of Ghana were more positive about service quality than those in the Ashanti region and suggested that the implementation of capitation payment in the Ashanti region may have influenced the perceived quality of care delivery in that region. Our finding, furthermore, is consistent with findings from similar studies in Ghana [23, 24] and Iraq [25] but also at variance with those of others studies from Tanzania [26], Korea [27] and Ghana [2]. The differences in quality perception between Ghana and Korea may be attributed to the level of development in these two countries, considering that Korea’s health care system is much more advanced than that of Ghana and that the level of health care expectations of Korean citizens may be higher than that of Ghanaians. The differences in perception among different studies from Ghana and between those of Ghana and Tanzania may also be attributed to the respective study settings.

### Association between subscriber characteristics and perception of quality of healthcare

It was significant to note that ssubscribers with lower occupational class were less likely to perceive quality of care as good compared to those in senior or managerial positions, a finding that contradicts those of Dixon et al. [23] and Amo-Adjei et al. [2]. It was also instructive to find that subscribers with more years of enrolment in the scheme were less likely to rate their perception of quality of care as good compared to those newly enrolled onto the Scheme. A plausible explanation may be that the longer one stays as an insured member, the less additional satisfaction s/he gains from utilizing the services and this may also partially explain why NHIA reports low rate of renewals among the insured population. These findings also go to support findings from other studies [28–33] that show that perceptions of clients on quality healthcare are often influenced by attributes such as gender, age, cultural orientation, religion, geographic location (rural or urban) and income levels. Therefore, our findings, supported by findings from others studies, demonstrate that region of residence is another factor that influences subscribers perception of quality of care under the capitation payment policy in the Ashanti region.

### Association between care provider characteristics and quality of health care

With regard to provider characteristics and quality of health care, a substantial proportion of NHIS-credentialed providers expressed good perception of quality of care they deliver to patients, but unlike the subscribers, their perception differ significantly by region. Care providers in the Volta region were less likely to rate their perception of quality of care as good compared to those in the Ashanti region. This trend, however, is contrary to expectation that providers in the Ashanti would express relatively lower perception of quality considering the health care providers associations’ agitations that heralded the introduction of capitation payment in the Ashanti region. One variable that shows significant difference among providers, however, was type of health care facility they operate. Health care providers working in hospital level facilities were significantly more likely to perceive the quality of care as good compared to those working at the health centre levels. A plausible explanation may be the fact that hospital level facilities are better equipped with personnel and equipment that guarantee good quality service provision than the lower level facilities. Other low level facilities such as clinics were also more positive about the quality of care, compared with health centres, although not significant. This is not surprising because in the classification of facilities for credentialing by the NHIA, clinics come next to hospital level facilities and are mostly owned by private medical practitioners and have facilities that may compare favourably with personnel and equipment in hospital level facilities. Health Centres, on the other hand, are largely manned by Medical/Physician Assistants whose expectation of care delivery may not be met because of practice guidelines that limit the level of service they can provide in their facilities and therefore their low perception of quality may be influenced by the restrictions on their practice.

### Important components that influence people’s perception of quality of care

Results of our principal component analysis (CPA) showed that users of health care services placed more importance on communication, dignity and respect, accommodation and cleanliness and confidentiality instead of measures that reflect technical quality. This syncs with findings of a study in Nigeria in which “dignity” emerged as one of the important dimensions of perceived quality [34]. It is however, instructive to note that these five components accounted for about 65% of the total variance in both subscribers’ and providers’ perception of quality of care delivery, with communication obtaining the highest factor loadings in the case of providers and “dignity and respect” in the case of subscribers. Our analysis of the perception of quality components reveals that communication explains much of the perceived good quality of care expressed by both subscribers and healthcare providers; and this is not surprising as it corroborate findings in existing literature. Among the dimensions proposed by Lehtinen et al. [35] in their three-tier approach to service quality dimensions was inter-customer communication. Williams and Calnan [36] also reported in their study that patient satisfaction with care, among others, is strongly correlated with appropriate adequate communication with patients. Cohen [37] on his part, observed that among the main sources of patient dissatisfaction with quality of care is lack of opportunity on the part of the patients to ask questions about their ailment. Abramovitz et al. [38] also reported that explanation offered by nurses to patients was important determinant of patients’ satisfaction with quality of care. Atinga et al. [30] also found that among the three independent variables that were statistically significant in explaining patient overall satisfaction with quality services was communication. The high level of importance users of health care services give to non-technical quality dimension such as communication and provider-client inter-personal relationship point to their limited knowledge of what constitutes quality healthcare as suggested by Alhassan et al. [22]. A study in Burkina Faso [39] reported that even though insured clients rated quality healthcare dimensions high, these clients actually received lesser technical quality care. Alhassan et al. [22] on their part observed that the tendency for clients to respond favorably to questions on quality healthcare dimensions could be high but not necessarily reflect their experiences and judgment of the quality situation. Thus, the quality of patient-provider communication has a very strong significant influence on the patient’s overall perception of quality care delivery [31] and as (Fottler et al. [40] suggested, good quality of patient-provider communication offers a healing environment where the former is more likely to continue utilizing services provided by the latter. These are summed up in the assertion that proper communication offers some form of medicine to the patient since effective communication reduces anxiety [41]. This finding suggests that healthcare providers have good interactions with their clients during healthcare delivery and as noted by Alhassan et al. [22] this should encourage the NHIA to integrate non-technical quality care indicators into its mainstream post credentialing monitoring tools used for their routine monitoring of health care providers in order to promote client-centered quality care improvement alongside the technical quality care standards. It also becomes important for healthcare managers to constantly determine the factors associated with the satisfaction of patients with the quality of care provided so as to understand what is valued by the patient, how the quality of care is construed by the patient and to determine where, when and how service change and improvement may be made [42].

One limitation of our study is that it missed out on the strengths of qualitative study which would have allowed for further probing into the responses of respondents to better understand the reasons for respondents’ responses to the questionnaires administered for enhanced discussion of findings. Another limitation may be seen from the study design which is cross-sectional instead of a true step-wedged design before-after study as originally envisaged. We also acknowledge the difficulty in making comparison between the three regions, considering some differences in their demographic and socio-economic characteristic. However, the two control regions have similar demographic and socio-economic characteristics and the differences between them and the intervention region are not too significant. These limitations notwithstanding, since the subject under study is a policy that is intended to be rolled out across the country, with a roll out plan already determined, what this study seeks to do is to identify issues that need attention of policy makers to improve on the implementation process. Hence, findings from the study provide valuable insight that can aid the debate surrounding the capitation payment policy in Ghana.

## Conclusion

This study assessed the perception of quality of care provision among subscribers of the NHIS and NHIS-credentialed providers in the Ashanti region where capitation payment is being implemented and two other regions where capitation payment is yet to be implemented with the objective to determine whether capitation payment influenced the quality of care perception of respondents in the Ashanti region. Findings from the study show that respondents’ background characteristics influenced their perception of quality of care but we also found that although both subscribers and care providers across the three regions have relatively good perception of the quality of health care in general, the quality perception among subscribers in the Ashanti region was relatively lower, compared to that in the Central region. We, therefore, conclude that in addition to their background characteristics, capitation payment may have influenced people’s perception of quality of care in the Ashanti region. The NHIA may, therefore, want to address itself to the negative issues raised by the public about the capitation implementation, as well as other factors that influence people’s perception of quality of care that are documented in existing literature in order to improve the perception of quality care delivery in the Ashanti region in particular; and under the NHIS in general.

## Additional files

Additional file 1: Subscriber/household sample size determination. (DOCX 14 kb)

Additional file 2: NHIS-Credentialed provider interview questionnaires. (DOCX 201 kb)

Additional file 3: Proportion of variance explained by each component in subscribers’ perception of quality of care. (DOCX 21 kb)

Additional file 4: Proportion of variance explained by each component in healthcare providers’ perception of quality of care. (DOCX 20 kb)

## Abbreviations

DRG: Diagnosis-related grouping
EA: Enumeration area
EPI: Expanded programme on immunization
FFS: Fee-for-service
GHS: Ghana health service
GLSS: Ghana living standard survey
GSS: Ghana statistical service
NHIA: National health insurance authority
NHIS: National health insurance scheme
OPD: Out-patients’ department
PES: Post-enumeration survey
USA: United States of America
WHO: World Health Organization
