# Supplementary material for: Perception of quality health care delivery under capitation payment: a cross-sectional survey of health insurance subscribers and providers in Ghana
Source: BMC Fam Pract. 2018 Mar 7;19:37. doi: 10.1186/s12875-018-0727-4 (PMC5842640; doi:10.1186/s12875-018-0727-4)
Supplement: Supplementary file 1 — Subscriber/household sample size determination. (DOCX 14 kb) [file 12875_2018_727_MOESM1_ESM.docx]

**Additional file 1:** Subscriber/household sample size determination

| Region | Clusters per GSS PES design | Household/subject per cluster^*^ | Total sample size |
| --- | --- | --- | --- |
| Ashanti | 45 | 10 | 450 |
| Volta | 23 | 18 | 414 |
| Central | 19 | 21 | 399 |
|  | **87** |  | **1,263** |

^*Based on WHO Immunization coverage cluster survey reference manual^
