# Supplementary material for: Perception of quality health care delivery under capitation payment: a cross-sectional survey of health insurance subscribers and providers in Ghana
Source: BMC Fam Pract. 2018 Mar 7;19:37. doi: 10.1186/s12875-018-0727-4 (PMC5842640; doi:10.1186/s12875-018-0727-4)
Supplement: Supplementary file 2 — NHIS-Credentialed provider interview questionnaires. (DOCX 201 kb) [file 12875_2018_727_MOESM2_ESM.docx]

# PART THREE

**NHIS-CREDENTIALLED PROVIDER INTERVIEW**

| Title of Study: | Provider payment reforms within the National Health Insurance Scheme:  Monitoring and Evaluation of Capitation as a provider payment mechanism for primary out-patients services. |
| --- | --- |
| Principal Investigator: | Francis-Xavier Andoh-Adjei |
| Certified Protocol Number | 057/13-14 |

Section **B**– CONSENT TO PARTICIPATE IN RESEARCH

**General Information about Research**

We are conducting a study on the implementation of capitation as a provider payment mechanism in order to assess its effect on health service delivery at the primary out-patient department of NHIS-credentialed facilities and to explore NHIS card bearers’ perception of quality of service and how that perception influence their insurance membership status and health seeking behavior. We shall need about one hour of your time to respond to some questions that we will ask you for answers.

**Benefits/Risk of the study**

The results of the study will help the National Health Insurance Authority to improve its operations for the benefit of all stakeholders and the nation as a whole.

**Confidentiality**

We shall not disclose the identity of any person who responds to our questions or who provide us with any information that will help us in this research. Names of persons who respond to our questions will not be written in any of our reports or articles that will be published for international consumption.

Apart from the university authorities and the research team, no other persons will have access to the research records. The final results, which will be in the form of a report, will however be shared with all stakeholders, that is the NHIA, providers and subscribers at NHIS stakeholders’ forum. Articles will also be prepared from the reports and published in international peer-review journals.

**Compensation**

This is an academic study intended to help NHIA improve on its operations for the benefit of the entire society and therefore there will not be any incentive whatsoever for people who volunteer to respond to the questions that we shall ask.

**Withdrawal from Study**

Your participation in this study is voluntary and you are free to withdraw from the study or even refuse to answer any question if you wish and nobody will fault you for your decision to withdraw from the study or your refusal to answer any question.

**Contact for Additional Information**

In case you have any questions or information about this research that you may want to convey to us, you may direct them to the following: **Francis-Xavier Andoh-Adjei, Head of Planning, Monitoring & Evaluation and International Relations, National Health Insurance Authority, 36-6th Avenue, Ridge, PMB, Ministries Post Office, Accra. You can also reach him on telephone numbers: 024 461 3747 or 020 923 4905.**

Section C- VOLUNTEER AGREEMENT

**"I have read or have had someone read all of the above, asked questions, received answers regarding participation in this study, and am willing to give consent for me, my child/ward to participate in this study. I will not have waived any of my rights by signing this consent form. Upon signing this consent form, I will receive a copy for my personal records."**

Name of Volunteer:

Signature or mark of volunteer Date

**If volunteers cannot read the form themselves, a witness must sign here:**

I was present while the benefits, risks and procedures were read to the volunteer. All questions were answered and the volunteer has agreed to take part in the research.

Name of witness

Signature of witness Date

I certify that the nature and purpose, the potential benefits, and possible risks associated with participating in this research have been explained to the above individual.

Name of Research Assistant

Signature of Research Assistant Date

#### IDENTIFICATION

| NAME OF RESPONDENT: ................................................................................. | |  |
| --- | --- | --- |
| GENDER: (MALE=1, FEMALE=2) | |  |
| TELEPHONE NUMBER: .................................................................................... | |  |
| REGION: (ASHANTI=1, VOLTA=2, CENTRAL=3) | |  |
| DISTRICT: ................................................................................................. ............ | |  |
| ENUMERATION AREA BASE NAME ………………………………………… | |  |
| URBAN/RURAL (URBAN=1, RURAL=2) | |  |
| COMMUNITY (CITY=1, LARGE TOWN=2, SMALL TOWN=3 VILLAGE=4) | |  |
|  | |  |
| LANGUAGE OF QUESTIONNAIRES: **ENGLISH**  LANGUAGE OF INTERVIEW LANGUAGE OF RESPONDENT  WAS TRANSLATOR USED? (YES=1, NO=2)  ***LANGUAGE CODES: (ENGLISH=1, AKAN=2, GA=3, EWE=4, NZEMA=5,  DAGBANI=6, OTHER=7).........................................  SPECIFY | |  |
|  | |  |
| **NAME OF RESEARCH ASSISTANT** | **SIGNATURE** | **DATE** |
|  |  | DAY  MONTH RESULTS |
| NAME OF SUPERVISOR | SIGNATURE | DAY  MONTH  RESULTS |
| RESULTS CODES: COMPLETED=1, PARTIALLY COMPLETED=2, REFUSED3, OTHER=4 …………………  (SPECIFY) | | |

**SECTION 1: RESPONDENT’S BASIC INFORMATION**

**I would like to ask some questions about yourself and I shall be grateful for your ARESPONSES**

| **NO.** | **Question** | **Response** | **Response code** |
| --- | --- | --- | --- |
| 1. | How old were you at your last birthday? | AGE IN COMPLETE YEARS | ………………….. |
| 2. | What is your primary status at this facility? | 1. MEDICAL OFFICER 2. ADMINISTRATOR 3. NURSE-IN-CHARGE 4. MEDICAL ASSISTANT 5. OTHER(SPECIFY) |  |
| 3. | How many years have you been in this practice/employment? | IN COMPLETE YEARS | ………………….. |
| 4. | This facility is owned by … | 1. (QUASI)-GOVERNMENT 2. MISSION 3. PRIVATE   4. OTHER ……………………  SPECIFY |  |
| 5. | What type of facility do you work in / operate? | 1. CHPS COMPOUND   1. MATERNITY HOME 2. HEALTH CENTER 3. CLINIC 4. HOSPITAL |  |
| 6. | On the average, how many clients access services at this facility **per week**? | 1. ≤ 25  2. 26-50  3. 51-75  4. 76-100  5. > 100 |  |

#### SECTION 2: PROVIDER PREFERENCE FOR PAYMENT METHODS WITHIN THE NHIS

| **Now, I would like to find out your preference for a particular payment mechanism.** | | |
| --- | --- | --- |
| NO. | **QUESTION** | **RESPONSE** |
| 7. | Which of the provider payment mechanism do you prefer for the following services that you provide to NHIS-insured members?: |  |
| a. | Primary out-patient services | 1. FEE-FOR-SERVICE 2. G-DRG 3. CAPITATION |
| b. | Non-primary out-patient services | 1. FEE-FOR-SERVICE 2. G-DRG 3. CAPITATION |
| c. | In-patient services | 1. FEE-FOR-SERVICE 2. G-DRG 3. CAPITATION |
| d. | Medicines  **(This refers to medicines dispensed at the out-patient department)** | 1. FEE-FOR-SERVICE 2. G-DRG 3. CAPITATION |
| e. | Emergencies | 1. FEE-FOR-SERVICE 2. G-DRG 3. CAPITATION |
| f. | Referral cases | 1. FEE-FOR-SERVICE 2. G-DRG 3. CAPITATION |
| g. | Diagnostics | 1. FEE-FOR-SERVICE 2. G-DRG 3. CAPITATION |
| 8. | Give no more than **4 key reasons** for the payment method you prefer most. | 1.  2.  3.  4. |

#### SECTION 3: QUALITY OF CARE

| **Now, I would like to know about your (facility’s) operations in the past one year with particular reference to the year January 1st 2013 and ending December 31st 2013 and I shall be very grateful for your kind responses.** | | |
| --- | --- | --- |
| **Access (to facility, diagnostics, medicines,)+( co-payments)** | | |
| 9. | What time do you start work at your facility? |  |
| 10. | What time do you close work at your facility? |  |
| 11. | Do you provide (emergency) services on week-ends? | 1. YES 2. NO |
| 12. | Does your facility offer laboratory services? | 1. YES  **2.** NO (**If “NO” go to 14)** |
| 13. | What proportion of cases that require laboratory investigations do you refer to the lab before prescribing treatment for the clients? | 1. 0% - 10% 2. 11% - 20% 3. 21% - 30% 4. 31% - 40% 5. 41% - 50% 6. 51% - 60% 7. 61% - 70% 8. 71% - 80% 9. 81% - 90% 10. 91% - 100% |
| 14. | Are there any medicines within the benefits package that insured clients may require but which you were not able to provide in your facility in the past 3 months? | 1. YES 2. NO |
| 15. | Which of these medicines were you not able to provide in your facility | 1. …………………………………. |
|  | Facility (**maximum top 3 medicines**)? |  |
|  |  | 2. …………………………………. |
|  |  | 3. …………………………………. |

| 16. | Give reasons why you are not able to provide those medicines. | | 1. NOT AVAILABLE. 2. PRICES HIGHER THAN NHIA TARIFFS   3. OTHER …………………….  SPECIFIY | |
| --- | --- | --- | --- | --- |
| 17. | Do you charge the clients for the cost beyond the tariff/capitated rate that the NHIA pays to you? | | 1. YES 2. NO | |
| 18. | On the average, how much time do you spend with a patient during consulting? | | 1. ≤ 5 MINS 2. 5-10 MINS 3. 10-15 MINS 4. >15 MINS | |
| Now, I would like to find out your opinion about the quality of primary care provision in your facility and I shall be grateful for your responses. The opinions are expressed in statements and your response will be to agree or dis-agree with the statement in the following rankings:  **Strongly agree (4) Agree (3) Dis-agree (2) Strongly dis-agree (1) Don’t know (8)** | | | | |
| **Staff availability** | | | | |
| 19. | There is always a prescriber at post to take care of clients anytime they come for treatment. | \| **4** \| **3** \| **2** \| **1** \| **8** \| \| --- \| --- \| --- \| --- \| --- \| | |  |
| 20. | We have enough prescribers to look after our clients in good time so they don’t wait too long at the facility. | \| **4** \| **3** \| **2** \| **1** \| **8** \| \| --- \| --- \| --- \| --- \| --- \| | |  |
| **Responsiveness** | | | | |
| 21. | The Doctors show interest in our clients and make them feel comfortable in our facility. | \| **4** \| **3** \| **2** \| **1** \| **8** \| \| --- \| --- \| --- \| --- \| --- \| | |  |
| 22. | The Nurses show interest in our clients and make them feel comfortable in our facility. | \| **4** \| **3** \| **2** \| **1** \| **8** \| \| --- \| --- \| --- \| --- \| --- \| | |  |
| 23. | Our consulting rooms are good enough to give privacy to clients when discussing their condition with the prescriber. | \| **4** \| **3** \| **2** \| **1** \| **8** \| \| --- \| --- \| --- \| --- \| --- \| | |  |

| 24. | We give our clients the opportunity to ask us question that they may have about the treatment we give before they leave our facility. | \| **4** \| **3** \| **2** \| **1** \| **8** \| \| --- \| --- \| --- \| --- \| --- \| |  |
| --- | --- | --- | --- | --- | --- | --- | --- | --- |
| **Accommodation/Cleanliness** | | | |
| 25. | Our facility has enough seats in the waiting area to seat all the clients who come for treatment. | \| **4** \| **3** \| **2** \| **1** \| **8** \| \| --- \| --- \| --- \| --- \| --- \| |  |
| 26. | Our environment, including the toilet facilities, is always neat. | \| **4** \| **3** \| **2** \| **1** \| **8** \| \| --- \| --- \| --- \| --- \| --- \| |  |
| 27. | Our waiting area is well ventilated. | \| **4** \| **3** \| **2** \| **1** \| **8** \| \| --- \| --- \| --- \| --- \| --- \| |  |
| **Service quality** | | | |
| 28. | We do proper diagnosis to ensure that we treat the condition effectively. | \| **4** \| **3** \| **2** \| **1** \| **8** \| \| --- \| --- \| --- \| --- \| --- \| |  |
| 29. | We explain to the clients how to take their medication and advise them on possible side effects. | \| **4** \| **3** \| **2** \| **1** \| **8** \| \| --- \| --- \| --- \| --- \| --- \| |  |
| 30. | We make time to advise our clients on disease. | \| **4** \| **3** \| **2** \| **1** \| **8** \| \| --- \| --- \| --- \| --- \| --- \| |  |

#### SECTION 4: PROVIDER PERCEPTION OF EFFECTS OF CAPITATION

| I would like to find out about your perception of the effects of capitation and I shall be grateful for your responses. The perceptions that you express will help the researcher to make recommendations to the NHIA for further improvement of the system. The opinions are expressed in statements and your response will be to agree or dis-agree with the statement in the follow rankings:  **Strongly agree (4) Agree (3) Dis-agree (3) Strongly dis-agree (2) Don’t know (8)** | | | | |
| --- | --- | --- | --- | --- |
| **Utilization of primary care services** | | | | |
| 31. | Capitation is a good way of eliminating provider shopping among the insured. | | \| **4** \| **3** \| **2** \| **1** \| **8** \| \| --- \| --- \| --- \| --- \| --- \| |  |
| 32. | Capitation has helped to minimize the multiple attendances by NHIS subscribers. | | \| **4** \| **3** \| **2** \| **1** \| **8** \| \| --- \| --- \| --- \| --- \| --- \| |  |
| 33. | Capitation helps to minimize the over-crowding of patients at the OPD. | | \| **4** \| **3** \| **2** \| **1** \| **8** \| \| --- \| --- \| --- \| --- \| --- \| |  |
| 34. | Capitation can create incentives for providers to reduce the quantity of service provided to the insured. | | \| **4** \| **3** \| **2** \| **1** \| **8** \| \| --- \| --- \| --- \| --- \| --- \| |  |
| **Quality of care/service (*Not applicable to Volta and Central*)** | | | | |
| 35. | | Capitation will help to improve the referral system in healthcare delivery because subscribers will all have to pass through the PHC system first. | \| **4** \| **3** \| **2** \| **1** \| **8** \| \| --- \| --- \| --- \| --- \| --- \| |  |
| 36. | | Capitation will lead to continuity of care because the insured client has only one provider that s/he sees anytime (s) he seeks treatment. | \| **4** \| **3** \| **2** \| **1** \| **8** \| \| --- \| --- \| --- \| --- \| --- \| |  |
| 37. | | Capitation will encourage referrals of potentially primary care cases to higher levels of care. | \| **4** \| **3** \| **2** \| **1** \| **8** \| \| --- \| --- \| --- \| --- \| --- \| |  |
| 38. | | Capitation can create incentives for the provider to reduce the quality of service provided to the insured. | \| **4** \| **3** \| **2** \| **1** \| **8** \| \| --- \| --- \| --- \| --- \| --- \| |  |

| **Workload and efficiency in service delivery (*Not applicable to Volta and Central*)** | | | | |
| --- | --- | --- | --- | --- |
| 39. | | Capitation has relieved us of the burden of OPD claims processing and submission. | \| **4** \| **3** \| **2** \| **1** \| **8** \| \| --- \| --- \| --- \| --- \| --- \| |  |
| 40. | | Capitation has helped to reduce our workload at the OPD. | \| **4** \| **3** \| **2** \| **1** \| **8** \| \| --- \| --- \| --- \| --- \| --- \| |  |
| 41. | | Capitation is contributing to efficiency in service delivery | \| **4** \| **3** \| **2** \| **1** \| **8** \| \| --- \| --- \| --- \| --- \| --- \| |  |
| **Cost containment (*Not applicable to Volta and Central*)** | | | | |
| 42. | Capitation provides incentives for us to manage our resources efficiently. | | \| **4** \| **3** \| **2** \| **1** \| **8** \| \| --- \| --- \| --- \| --- \| --- \| |  |
| 43. | Capitation enables us to do efficient purchasing of items | | \| **4** \| **3** \| **2** \| **1** \| **8** \| \| --- \| --- \| --- \| --- \| --- \| |  |
| 44. | Capitation will slow down growth in service utilization. | | \| **4** \| **3** \| **2** \| **1** \| **8** \| \| --- \| --- \| --- \| --- \| --- \| |  |
| 45. | Capitation can reduce NHIA’s expenditure on primary out-patients claims. | | \| **4** \| **3** \| **2** \| **1** \| **8** \| \| --- \| --- \| --- \| --- \| --- \| |  |
| **Income stability and financial planning (*Not applicable to Volta and Central*)** | | | | |
| 46. | Capitation has helped to eliminate the delayed reimbursement that is experienced under the G- DRG. | | \| **4** \| **3** \| **2** \| **1** \| **8** \| \| --- \| --- \| --- \| --- \| --- \| |  |
| 47. | Capitation provides a stable income for the provider because of the advance payment. | | \| **4** \| **3** \| **2** \| **1** \| **8** \| \| --- \| --- \| --- \| --- \| --- \| |  |
| 48. | Capitation helps us to plan our cash flow better than before. | | \| **4** \| **3** \| **2** \| **1** \| **8** \| \| --- \| --- \| --- \| --- \| --- \| |  |
|  | **Adequacy of the capitated rate (*Not applicable to Volta and Central*)** | | | |
| 49. | The capitated rate is enough to cover the primary OPD expenses on the insured clients. | | \| **4** \| **3** \| **2** \| **1** \| **8** \| \| --- \| --- \| --- \| --- \| --- \| |  |
| 50. | Capitation will reduce the income of the provider. | | \| **4** \| **3** \| **2** \| **1** \| **8** \| \| --- \| --- \| --- \| --- \| --- \| |  |
| 51. | Capitation will create incentive for the provider to pass on the extra cost of providing care to the insured client | | \| **4** \| **3** \| **2** \| **1** \| **8** \| \| --- \| --- \| --- \| --- \| --- \| |  |

| **Subscriber satisfaction/trust of primary care provider under Capitation ( *Not applicable to Volta and***  ***Central*)** | | | |
| --- | --- | --- | --- |
| 52. | Because the clients are tied to one provider, they do not feel happy with the services we provide to them. | \| **4** \| **3** \| **2** \| **1** \| **8** \| \| --- \| --- \| --- \| --- \| --- \| |  |
| 53. | They also are not get satisfied with the medication we give to them. | \| **4** \| **3** \| **2** \| **1** \| **8** \| \| --- \| --- \| --- \| --- \| --- \| |  |
| 54. | Because of capitation, the insured clients do not trust the quality of service that we provide to them. | \| **4** \| **3** \| **2** \| **1** \| **8** \| \| --- \| --- \| --- \| --- \| --- \| |  |

**We have come to the end of our questioning and we thank you very much for your time. If we require further information we will come back to you.**
