# Supplementary material for: Perception of quality health care delivery under capitation payment: a cross-sectional survey of health insurance subscribers and providers in Ghana
Source: BMC Fam Pract. 2018 Mar 7;19:37. doi: 10.1186/s12875-018-0727-4 (PMC5842640; doi:10.1186/s12875-018-0727-4)
Supplement: Supplementary file 3 — Proportion of variance explained by each component in subscribers’ perception of quality of care. (DOCX 21 kb) [file 12875_2018_727_MOESM3_ESM.docx]

**Additional file 3:** Proportion of variance explained by each component in subscribers’ perception of quality of care

| Component | Initial Eigenvalues | | | Extraction Sums of Squared Loadings | | |  | | |
| --- | --- | --- | --- | --- | --- | --- | --- | --- | --- |
|  | Total | % of Variance | Cumulative % | Total | % of Variance | Cumulative % | Total | % of Variance | Cumulative % |
| 1 | 4.728 | 26.269 | 26.269 | 4.728 | 26.269 | 26.269 | 3.415 | 18.973 | 18.973 |
| 2 | 2.808 | 15.599 | 41.868 | 2.808 | 15.599 | 41.868 | 2.803 | 15.572 | 34.545 |
| 3 | 1.550 | 8.609 | 50.477 | 1.550 | 8.609 | 50.477 | 2.194 | 12.189 | 46.734 |
| 4 | 1.401 | 7.785 | 58.262 | 1.401 | 7.785 | 58.262 | 1.721 | 9.561 | 56.295 |
| 5 | 1.143 | 6.353 | 64.614 | 1.143 | 6.353 | 64.614 | 1.497 | 8.319 | 64.614 |
| 6 | .919 | 5.104 | 69.719 |  |  |  |  |  |  |
| 7 | .786 | 4.367 | 74.086 |  |  |  |  |  |  |
| 8 | .745 | 4.138 | 78.224 |  |  |  |  |  |  |
| 9 | .671 | 3.729 | 81.954 |  |  |  |  |  |  |
| 10 | .656 | 3.646 | 85.600 |  |  |  |  |  |  |
| 11 | .551 | 3.058 | 88.658 |  |  |  |  |  |  |
| 12 | .503 | 2.795 | 91.454 |  |  |  |  |  |  |
| 13 | .470 | 2.608 | 94.062 |  |  |  |  |  |  |
| 14 | .342 | 1.903 | 95.965 |  |  |  |  |  |  |
| 15 | .276 | 1.534 | 97.499 |  |  |  |  |  |  |
| 16 | .257 | 1.428 | 98.927 |  |  |  |  |  |  |
| 17 | .175 | .974 | 99.901 |  |  |  |  |  |  |
| 18 | .018 | .099 | 100.000 |  |  |  |  |  |  |
