# Supplementary material for: Perception of quality health care delivery under capitation payment: a cross-sectional survey of health insurance subscribers and providers in Ghana
Source: BMC Fam Pract. 2018 Mar 7;19:37. doi: 10.1186/s12875-018-0727-4 (PMC5842640; doi:10.1186/s12875-018-0727-4)
Supplement: Supplementary file 4 — Proportion of variance explained by each component in healthcare providers’ perception of quality of care. (DOCX 20 kb) [file 12875_2018_727_MOESM4_ESM.docx]

**Additional file 4:** Proportion of variance explained by each component in healthcare providers’ perception of quality of care

| Component | Initial Eigenvalues | | | Extraction Sums of Squared Loadings | | | Rotation Sums of Squared Loadings | | |
| --- | --- | --- | --- | --- | --- | --- | --- | --- | --- |
|  | Total | % of Variance | Cumulative % | Total | % of Variance | Cumulative % | Total | % of Variance | Cumulative % |
| 1 | 5.310 | 44.250 | 44.250 | 5.310 | 44.250 | 44.250 | 4.163 | 34.695 | 34.695 |
| 2 | 1.389 | 11.575 | 55.825 | 1.389 | 11.575 | 55.825 | 2.075 | 17.291 | 51.986 |
| 3 | 1.144 | 9.531 | 65.356 | 1.144 | 9.531 | 65.356 | 1.604 | 13.370 | 65.356 |
| 4 | .941 | 7.840 | 73.196 |  |  |  |  |  |  |
| 5 | .806 | 6.718 | 79.915 |  |  |  |  |  |  |
| 6 | .647 | 5.388 | 85.303 |  |  |  |  |  |  |
| 7 | .505 | 4.212 | 89.515 |  |  |  |  |  |  |
| 8 | .430 | 3.580 | 93.095 |  |  |  |  |  |  |
| 9 | .319 | 2.660 | 95.755 |  |  |  |  |  |  |
| 10 | .228 | 1.904 | 97.659 |  |  |  |  |  |  |
| 11 | .172 | 1.435 | 99.094 |  |  |  |  |  |  |
| 12 | .109 | .906 | 100.000 |  |  |  |  |  |  |
| Extraction Method: Principal Component Analysis. | | | | | | | | | |
